# The re-emergence and transmission of Monkeypox virus in Nigeria: the role of one health

**DOI:** 10.3389/fpubh.2023.1334238

**Published:** 2024-01-05

**Authors:** Seto C. Ogunleye, Olalekan C. Akinsulie, Abdullahi T. Aborode, Mercy M. Olorunshola, Damilola Gbore, Moyinoluwa Oladoye, Ridwan O. Adesola, Joy O. Gbadegoye, Boluwatife J. Olatoye, Mariam A. Lawal, Akeem B. Bakare, Oluwabori Adekanye, Ezemba C. Chinyere

**Affiliations:** ^1^Faculty of Veterinary Medicine, University of Ibadan, Ibada, Nigeria; ^2^Department of Veterinary Biochemistry, Nigeria College of Veterinary Medicine, University of Ibadan, Ibada, Nigeria; ^3^Healthy Africans Platform, Research and Development, Ibada, Nigeria; ^4^Department of Pharmaceutical Microbiology, Pharmacy, University of Ibadan, Ibada, Nigeria; ^5^Department of Biochemistry, Department of Biochemistry, Federal University of Agriculture, Abeokuta, Nigeria; ^6^University of Ibadan, Ibada, Nigeria; ^7^Chukwuemeka Odumegwu Ojukwu University, Uli, Nigeria

**Keywords:** Monkeypox virus, disease transmission, zoonosis, one health, epidemiology

## Abstract

The Monkeypox virus, commonly abbreviated as mpox, is a viral zoonosis that is experiencing a resurgence in prevalence. It is endemic to regions of West and Central Africa that are characterized by dense forested areas. Various measures pertaining to animals, humans, and the environment have been recognized as potential factors and catalysts for the spread of the disease throughout the impacted regions of Africa. This study examines the various factors contributing to the transmission of the virus in Nigeria, with a particular focus on the animal-human and inter-human modes of transmission in rural communities and healthcare facilities. The One Health approach was emphasized as crucial in the prevention and management of this issue. Literature suggests that preventing repeated zoonotic introductions could potentially halt the transmission of the mpox virus from animal to human hosts, leading to a potential decrease in human infections.

## 1 Introduction

The mpox virus is classified as a double-stranded DNA orthopoxvirus within the Poxviridae family. It is responsible for the outbreak of monkeypox in various populations worldwide. This information is supported by reference (1). The monkeypox virus is characterized by an incubation period ranging from 5 to 21 days, which is then followed by a series of symptoms including fever, rash caused by cellulitis, sore throat, lymphadenopathy, secondary bacterial infections, respiratory distress, bronchopneumonia, gastrointestinal involvement, dehydration, sepsis, encephalitis, and corneal infection that may lead to loss of eyesight. Since the initial detection of the initial human case in 1970, there has been a rise in the prevalence of monkeypox, with notable occurrences observed in Nigeria, the Democratic Republic of the Congo, Liberia, sCameroon, Gabon, Sudan, Sierra Leone, the Republic of the Congo, and the Central African Republic (1–7). The transmission cycle of mpox virus entails the active involvement of wild animals, including rope squirrels, tree squirrels, and Gambian pouched rats, which have been identified as susceptible to the virus. This highlights the zoonotic nature of mpox virus.

There are various factors that facilitate the transmission and establishment of the disease caused by mpox virus. These factors can be broadly categorized into agent, host, and environmental-related drivers, and include anthropogenic activities and biosecurity challenges. The present method of diagnosing involves the identification of clinical manifestations, employment of transmission electron microscopy, Polymerase Chain Reaction (PCR), tissue culture, immunofluorescence assay, and enzyme-linked immunosorbent assay (ELISA) (8). Currently, prevention strategies primarily rely on hygienic practices and limitations to wild contacts. However, significant progress has been made in the development of vaccines (9).

## 2 Agent related factors

### 2.1 Monkeypox virus

**Figure 1:**
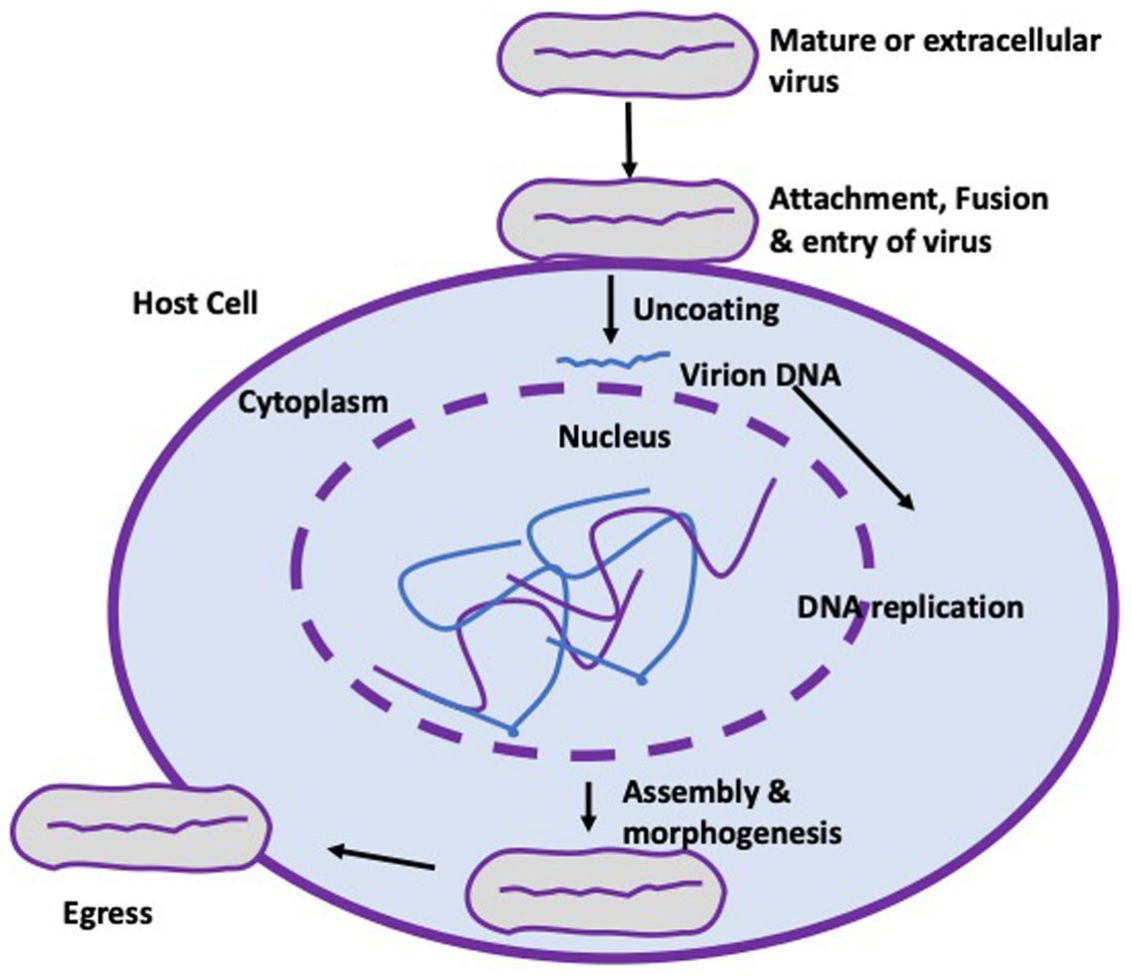
Life cycle of the mpox virus. The virus attaches to the cell membrane of a target cell with the aid of Toll-like receptors (not shown), enabling fusion and entry of the virus. Uncoating and replication of the virus takes place strictly in the cytoplasm of infected cells before assembling, morphogenesis and eventual egress from the cell to form an extracellular enveloped virion.

The genome of the mpox virus is composed of a double-stranded linear DNA enclosed by a lipoprotein membrane. This virus is capable of adapting to its environment and is known to induce symptoms similar to those of smallpox in humans upon infection. The virus is typically recognized for its substantial size, measuring approximately 200–250 nanometers, and its brick-like or ovoid morphology, as documented in sources 11 and 12. The virus necessitates the utilization of cellular components, particularly ribosomes, from their infected hosts for the purpose of translating mRNA, despite the existence of a strong viral cellular component for replication, transcription, assembly, and egress, as depicted in Figure 1. Following the successful eradication of the smallpox virus in 1970, the mpox virus emerged as the most prevalent and virulent orthopoxvirus in the human population. Despite its limited transmission and tendency toward short-lived epidemics, the virus has emerged as a significant global health threat since its initial identification as the causative agent of monkeypox in the Democratic Republic of Congo in 1970. The resurgence of monkeypox, observed in Nigeria and other nations, is hypothesized to be linked to the discontinuation of smallpox vaccination in 1980. This phenomenon may be attributed to a decrease or absence of population immunity toward the poxviruses (10).

This is corroborated by the prevalence of drivers, such as the significant surge of monkeypox virus cases in regions where the disease is endemic within the country. Additional factors encompass the worldwide escalation of economic, political, and cultural interconnections, alterations in climate patterns, and the lack of proactive monitoring for the monkeypox virus within Nigeria. The mpox virus is classified into two distinct clades: the Congo Basin clade, which is characterized by a high transmission rate and a case fatality rate of approximately 10%, and the West African clade II, which is associated with limited human-to-human transmission and a lower case-fatality rate of less than 1%, although recent outbreaks in Nigeria have challenged this observation (11–13).

### 2.2 Transmission and spread

**Figure 2:**
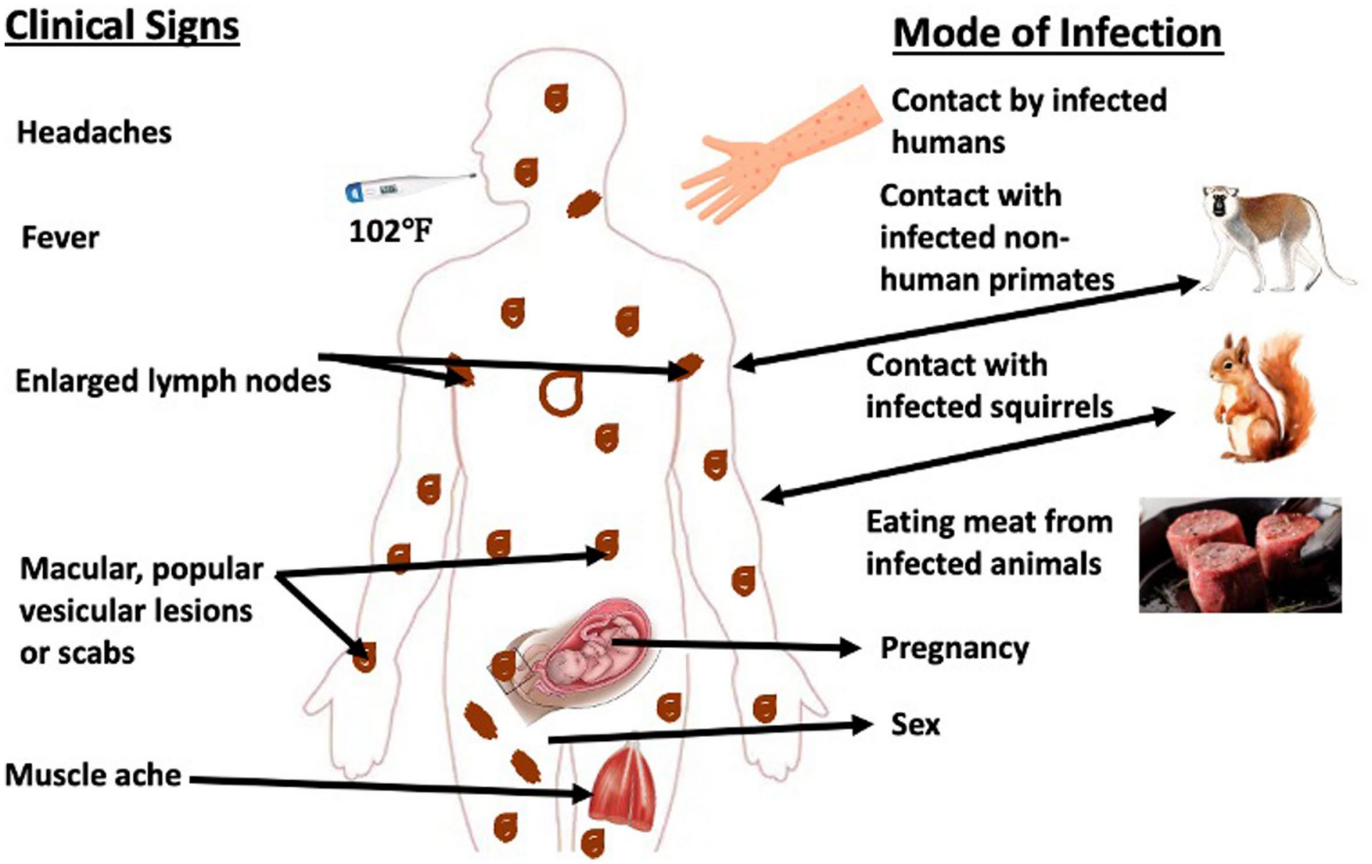
Schematic representation of the transmission cycle of mpox virus. Typically, mpox virus is transmitted via contact with infected animal or human hosts. Moreover, there have been evidences of *in utero* and sexual transmission of the virus. The clinical signs associated with the infection range from high fever, severe headache to muscular lesions with pains, and lymphangitis.

The transmission of mpox virus into human populations is known to be facilitated by wild animals, including squirrels, sooty mangabey (*Cercocebus atys*), and Gambian rats, which play significant roles in this process (14, 15). The primary modes of transmission to the human population are through animal-to-human and human-to-human contact (10). The transmission of a pathogen from animals to humans can take place through various means, including direct contact with an infected or carrier animal, inhalation of aerosols, consumption of infected host animal, and exposure to bodily fluids such as respiratory droplets and blood (as illustrated in Figure 2). The mode of transmission of the virus involves its entry into the human body through the respiratory tract, broken skin, or mucous membranes such as the eyes, nose, or mouth.

The likelihood of zoonotic transmission from animals to humans is elevated in instances of physical contact such as scratches, bites, injuries sustained during the preparation of bush meat, or through direct or indirect exposure to bodily fluids or lesion materials. Transmission of the disease can be attributed to occupational exposure, tourism, or a compromised interface between humans and animals (14–16). The Democratic Republic of Congo’s earliest case was historically attributed to a hunter who killed a squirrel exhibiting symptoms and pox-like lesions on its body. Similarly, in Côte d’Ivoire, the earliest case was discovered at the Tai National Park when a sooty mangabey was found deceased with pox-like (17, 18). The transmission of the virus from one human to another occurs through contact with respiratory droplets, lesions, body fluids, and contaminated personal objects that provide a suitable environment for viral replication (14).

Moreover, it has been verified that transmission within the hospital has occurred, while the transmission through sexual contact is presumed in the patient exhibiting genital lesions caused by the mpox virus. The transmission of congenital monkeypox is currently understood to occur through two primary modes: vertical transmission via the placenta from mother to child, and perinatal transmission during childbirth when neonates come into contact with pox lesions located on the genitalia. This information is supported by previous research (19, 20).

### 2.3 Pathogenesis and mutations

Mpox virus characteristically has incubation period of 5–20 days and are known to replicate at the site of inoculation following the oropharynx, nasopharynx, or intradermal routes of entry into its susceptible hosts. This is usually followed by the spread from site of entry to regional lymph nodes and subsequent viremia before spread to other part of the body organs. Mpox virus enters host cell via induction of micropinocytosis and fusion where it utilizes the cytoplasmic nutrients of the host cells to complete the life cycle and replication (21–23).

Previously, it is believed that the mpox virus does not show a record of mutations compared to the HIV or SARS-CoV-2 (12). However, this belief has been refuted by the current global outbreaks with most of the isolated monkeypox viruses showing more mutations, not less than 40, attributable to current trends in transmission evolution and dynamics among several host species (12, 24). Interestingly, there is currently insufficient evidence to prove how mpox mutants interacts with the host and the implication of the mutations on its replication, hence the needs for more scientific exploration, especially into the dynamism and complexity of the virus.

### 2.4 Immune response

Poxviruses have created a variety of methods to subvert, hijack or exacerbate the host immune response to infection (25). Several research has been done on the pathophysiology and pathogenesis of mpox virus, but the innate and adaptive immune responses to the infection are still poorly known due to a lack of data (26). Neutralizing antibodies and residual IgG have been linked to milder illness in infected patients (27) and were demonstrated to persist in vaccine recipients (7, 28, 29). However, there is no information available about T cell and antibody responses to human mpox virus infections among the Nigerian population. Currently, there are no records or studies on the immune responses that occur after experimental infections in animal models (26). The later could be an interesting research area to explore in the future.

## 3 Host related factors

### 3.1 Smallpox vaccination regimen

Several factors have been linked to the sudden re-emergence of the mpox in Nigeria since 2017 after the last reported case in 1978. Prior to the 2017 epidemic in Nigeria, most human cases of monkeypox occurred in rural, forested regions of Africa; however, cases have occurred in urban settings, suggesting new risk factors could be potentiating this re-emergence. The cessation of the administration of the smallpox vaccine in Nigeria since 1980 could be a critical factor enhancing the re-emergence of mpox virus among individuals previously vaccinated with smallpox vaccine, due to waning immunity against mpox virus. Additionally, declination of herd immunity could be another ripple effect of the end of smallpox vaccination.

### 3.2 Human-animal interphase

Mpox virus outbreaks in the human population in various regions of Nigeria have been directly linked to consumption of wild animals such as giant rats and monkeys (Figure 2). Numerous species of small mammals, rats, and primates can be found in Southern Nigeria’s mangroves and tropical rainforest, where most of the recent outbreaks occurred (30). Most southern states like Rivers, Bayelsa, Lagos, and Delta were implicated in the outbreak (Figure 2). However, this might not reflect the true extent of the disease’s burden in Nigeria as factors like the disease underreporting and concealment by infected people due to fear of stigmatization might have confounded the exact picture.

Moreover, poor systemic surveillance complicated by the Covid-19 pandemic at the time when the data was collected could also reduce the true extent of the situation (31, 32). The growing contact between people and small mammals that could be carrying the mpox virus is another major element that is thought to contribute to the prevalence of mpox in Nigeria (33). Civil wars, refugee displacement, farming, deforestation, climate change, demographic changes, and population movement may have led to a spread of monkeypox-infected animals and increased their interaction with humans across West and Central Africa (33).

The possibility of an epizootic event straddling the Nigeria–Cameroon border was raised in 2008 when one mpox sample isolated from a case-patient in Cameroon was discovered to be genetically similar to a sample from Nigeria despite no epidemiologic association (34). The fact that some outbreak had disproportionately more men than women underscore the fact that men tend to engage in the majority of high-risk activities with wild animals, such as hunting and bush meat trade (35, 36). Healthcare professionals, who are likely to have contact with infected animals and humans, are potentially at risk of getting infected with mpox virus. Seasonal diversity of mpox virus is suggested to be evident with higher outbreaks during the fall season owing to increased rainfall underscored by flooding, deforestation, increased farming, and hunting activities, increased anthropogenic activities etc.

These factors cause increased break in human-animal interface, relocation of animals from the wild to human population and residences and increased human-wild contacts. Thereby, increasing the chances of spread of the virus to human population. The current global outbreaks stemming from 2018 and 2019 in West African outside the shores of Africa are caused by the West African clade based on genomic sequence and transcriptomic analysis (37, 38). Put together, the more interaction humans have with animals, the greater the risk of acquiring mpox virus.

### 3.3 Health status

Presence of a co-infection has been shown to affect the mpox disease progression in infected individuals. In epidemics, case fatality rates have ranged between 1 and 10%, with young adults and children dying most frequently. Those who have immunosuppression are especially vulnerable to serious illness. Up to 90% of patients experience lymphadenopathy, which is thought to be a clinical characteristic that separates human monkeypox from smallpox (39). According to a mathematical model investigating the connection between HIV and mpox virus coinfection, HIV may encourage the spread of the monkeypox virus and vice versa (33). A cross talk between HIV and monkeypox virus has also been reported (40). Nine HIV-1-coinfected patients were found in a cohort of 40 patients from hospital records in Nigeria to have larger skin lesions, more severe illnesses, and greater incidences of genital ulcers and bacterial superinfection than HIV-negative cases (2). Transmission of mpox vrius through sexual contact has also been suggested among cases in Nigeria similar to several other reports especially among gay, bisexual, and other men who have sex with men (GBMSM) (15, 20).

### 3.4 Symptoms

Monkeypox is generally believed to be a self-limiting illness with an incubation period commonly between 7 and 14 days but may take up to 21 days (41). After the appearance of fever, it is characterized by development of a distinctive rash that often begins on the face and spreads to other parts of the body which can associated with the initial reactions caused by the entry of the virus. Afterwards, headaches, muscle aches, backache, fatigue with progression to exhaustion and lymphadenopathy are symptoms that are commonly known to develop. Lesions characteristic of monkeypox principally starts within the oropharynx and then appear throughout the body which strongly supports the pathogenesis and progression of the virus in the host body. The current mortality rate ranges between 1 and 10% based on the clade of the infecting monkeypox virus strain, the immune and/or vaccination status of the host, and the availability of health care in the locality (41, 42).

### 3.5 Immunity

Smallpox vaccine has been shown to produce 85% immunity in vaccinated individuals and the CDC recommended getting vaccinated against smallpox within 2 weeks, ideally before 4 days following significant, unprotected contact with an infected animal or a confirmed human case (33). However, for severely immunocompromised individuals to whom the smallpox vaccine should not be administered, the vaccinia immune globulin can be utilized as prophylaxis. Tecovirimat and Brincidofovir, which can also be used to treat monkeypox, are antiviral medications that have been licensed by the Food and Drug Administration (FDA) to treat smallpox though there are no antiviral medications specifically designed to treat or prevent monkeypox. The two vaccines that have received FDA approval are ACAM2000 and JYNNEOS. ACAM2000 is the only vaccine that is advised for monkeypox post-exposure prophylaxis; JYNNEOS is a live, non-replicating virus that has been approved exclusively on monkeypox (33, 43).

## 4 Environmental factors

With the recent global outbreak of the mpox disease in regions that have not historically reported mpox virus, there is a need to pay close attention to the environmental factors that might be contributing to its transmission. The likelihood of zoonotic transmission is growing because of environmental factors increasing the frequency of contact with potential hosts.

### 4.1 Geographical location

Geographic ranges and local prevalence of mpox virus are projected to change as a result of climate change, either directly through impacts on the pathogen or indirectly through changes in the ranges of organisms that serve as vectors and reservoirs. A study has shown that climate is an important driving factor for the transmission of mpox virus from wildlife to humans (44). A previous study also suggests the association of mpox virus distribution with humid lowland tropical forests, high mean annual precipitation and low elevations (45). Similarly, temperature has been identified as a significant variable that contributes to mpox virus distribution in Africa (46). Hence, a good understanding of the geographic distribution of the disease may play a major role in constructing effective strategies in addressing the recent outbreaks.

### 4.2 Housing models

Residents of shared housing such as those found in prisons have been at-risk of human-to-human transmission of mpox virus (47). This agrees with another study that links the mpox virus transmission to poor housing structure (48). Like most infectious diseases, overcrowding and lack of social distancing amplifies the transmission of the mpox virus. Close contacts with infected persons are the primary mode of spread in human-to-human transmission. Furthermore, socio-economic factor remains a significant factor in the spread of monkeypox. A study reveals the correlation between low-income background and the incidence of mpox virus as more cases have been identified in such communities (49). A valid explanation to this might be that bush hunting being the predominant occupation among this population normally would expose them to the infected hosts more frequently.

### 4.3 International travels

International travel still seems to be a significant contributor to the spread of monkeypox as with other infectious diseases like COVID-19. Since mpox re-emerged in Nigeria in 2017, there have been isolated instances outside of Africa, either among people who have recently traveled to Nigeria or among secondary contacts of people with cases that are travel related. The first human monkeypox cases exported from Africa involved travelers from Nigeria to the United Kingdom (*n* = 2), Israel (*n* = 1), and Singapore (*n* = 1) (50). This has linked Nigeria to the initial sets of imported cases of monkeypox globally. However, a good number of the recent outbreaks seen in the Americas, Australia and Europe are not associated with direct travel history (51). This suggests a theory of communal mode of transmission.

### 4.4 Food safety

Food safety has always been an important branch of public health practice. This is true for zoonotic diseases that are associated with food substances (16). Given that a significant number of Nigerians who consume bush meat from wild animals as “delicacies” lack basic knowledge of the virus and how to safely and hygienically prepare the meat, it is possible that mpox disease is related to issues with food safety and cleanliness in the country (52). This necessitates the need to implement good hygiene and handwashing culture after interacting with wildlife and food animals generally (16).

## 5 Situation in Nigeria and the global community

**Figure 3:**
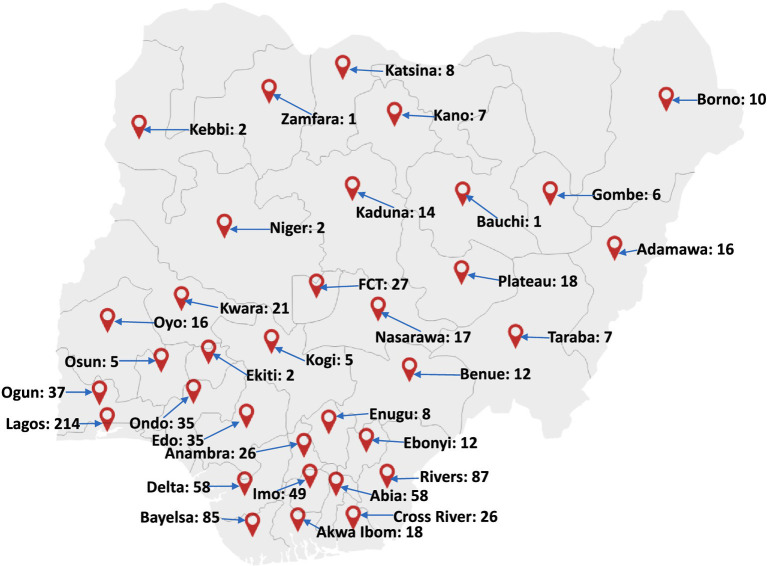
Map of Nigeria showing the distribution of mpox virus cases in Nigeria between 2017 and 2022. A large cluster of these cases were reported in the southern part of the country. Factors such as nearness to forests and animal-host population have been found to be major factors linked to the re-emergence and transmission of the pathogen. Adapted from Minhaj et al. (47).

Based on the available data and gaps between 1978 and 2017, the absence of reported cases might have been due to lack of testing, cases not being presented to the hospitals, lack of surveillance programs (53), incomplete reporting practices, and insufficient scientific research into mpox virus etc. This is evident in the number of cases reported between 2017 and 2022 (Figure 3) marked at approximately 558 suspected cases, 241 confirmed and 8 deaths spanning through all age groups as reported by the Nigeria CDC (54, 55). The current 2022 monkeypox virus outbreak has spanned through all the regions in Nigeria, presenting a more complicated conditions and needs for intensive efforts toward mitigating the spread of the virus as against the previous outbreaks.

Thanks to the advanced scientific approaches and collaborative efforts introduced for the surveillance of some other fatal viral epidemics such as Ebola fever, Lassa fever outbreaks and the COVID-19, proximity of residences to forests, which could drive zoonotic infections; and migration from rural to urban areas, which could drive inter-human transmissions (31, 32). The current land-use change, diversity and dynamics in Nigeria is worrisome and strongly portends strong change in the hazards of monkeypox spillover from the wild into the human population (38, 54).

Surprisingly, the current outbreak has not been able to have a defined primary source (s) of the virus in the non-endemic regions, and travel history especially from the endemic regions to the non-endemic regions are yet to be reported. One of the explanations to this current situation might be traceable to the onset of COVID-19 which impacted limitations to international travel and movement, consequently, resulting in the few cases of monkeypox virus cases reported in 2020 with no traceable travel record to endemic areas or mapped epidemiologically to West or Central Africa (38).

This strongly suggest human-to-human spread which possibly begun far before the current outbreaks; therefore, the disease has now become topical, indicating a possible community spread of the disease in an environment where it is not endemic and the urgent need for efforts toward mass human surveillance. As of now, there are approximately 21 confirmed cases of mpox virus with 1 death in Nigeria (51, 56, 57). The previous reports of the virus in Nigeria between 2017 and 2018 indicated that exported cases during the outbreak belonged to the West African clade and shared a common ancestor with a local case in Bayelsa State with a travel history to Bayelsa, Delta or Rivers State (38). This clade was found to show relatedness to the isolated cases in the UK, Israel, Singapore and Portugal. With African being the origin of the disease and core “exporter” to the developed nations evidenced by the transcriptomic analysis available till date, the current outbreaks are worrisome and may be pointer to the current spates of monkeypox virus cases in many other nations.

This is further reinforced by the current cessation of restriction imposed on COVID-19 enabling increased international travels. Although the only available data linking COVID-19 pandemic to monkeypox virus outbreak is the travel restriction which is believed to have lowered the spread of the virus, it is still unclear if there could be some form of genetic relatedness between the viruses responsible for these outbreaks. It is safe to propose that the current spate of the virus in Nigeria is motivated by the reduced herd immunity from smallpox vaccination which was identified to provide cross-protection for monkeypox virus (being genetically related, and bearing in mind that the effectiveness of smallpox vaccine in preventing monkeypox in humans is currently estimated to be about 85%, with some evidence toward the efficacy of ring vaccination to prevent transmission chains), increased human-wild contact including the sale and consumption of meat from the wild, poor community knowledge and lack of awareness of the disease, low efforts toward surveillance of the disease and limited financial support toward research and development. So far, not less than 21 countries have reported more than 270 monkeypox cases (178 confirmed and 92 suspected cases) with Spain (102), the UK (71) and Portugal (37) leading current case count (21).

Although the earliest cases reported in the UK between 2018 and 2022 were detected from individuals traveling from Nigeria, the current trends in the present-day outbreaks being reported outside Africa as distinctively demonstrated no sufficient epidemiological and phylogenetic evidences that has successfully linked the current global outbreak (beyond UK) of Monkeypox to Africa. The spate can be theoretically associated to the increased social interactions following the lifting of COVID-19 travel restrictions and international travels. This further suggests the paucity of surveillance of the virus, and subsequently, a strong justification for possible multiple outbreaks could be due to prior exportation of the virus from an endemic region followed by undetected chains of transmission into a non-endemic region. This could have therefore resulted in a substantial transmission in such naïve population and the current pockets of outbreaks. Transmission in the population can be enhanced by mass gatherings, increase contact rates among infected and susceptible individuals. The several outbreaks of monkeypox has involved the global response of WHO based on cases being reported from the UK (currently having over 240 cases), the Democratic Republic of Congo (DRC) with total reported cases of 10,459 suspected cases and 360 deaths (estimated case fatality (CFR) rate as 3.4%), Cameroon 25 cases and 3 deaths (CFR 8%), Central African Republic 6 cases and 2 deaths (CFR 33.3%) (57).

### 5.1 Limitations

Developing nations typically like Nigeria experience more severe impacts during an epidemic. Consequently, given that a majority of the countries afflicted by Monkeypox fall under this category, it is important to examine their principal challenges. Many urban areas experiencing rapid growth and development frequently have challenges in providing adequately equipped hospitals with the capacity to accommodate multiple patients simultaneously. Hospitals see rapid influxes of patients, leading to overwhelming conditions and limited capacity to provide treatment to all those seeking care. Furthermore, it is imperative to have access to high-quality equipment, such MRI scanners and oxygen tanks, as well as well-equipped laboratory facilities, inside hospital settings. Moreover, a considerable percentage of hospitals lack a designated isolation ward, which is a significant challenge given the highly transmissible nature of viral infections. Failure to mitigate the chain of transmission can lead to a rapid escalation in the number of patients. With the increasing number of cases, there is a growing demand for additional human resources and medical equipment.

Additionally, there exists a deficiency in diagnostic facilities for Monkeypox, with a notable scarcity of laboratories possessing the necessary capabilities to identify the disease. These laboratories are typically concentrated in urban areas and their availability is limited. This trend is a significant challenge as it predominantly impacts regions geographically distant from major urban centers, hence impeding the efficiency of testing procedures and imposing limitations on their scope. In addition to the issue of insufficient health infrastructures, communities provide a notable barrier due to the prevalence of distinct belief systems. A significant proportion of these communities attribute illnesses to supernatural forces and tend to prioritize seeking assistance from traditional healers over conventional medical interventions. In addition, it is important to consider the potential stigmatization that may compel those in need of medical aid to actively refrain from seeking such treatment, particularly while the virus continues to progress within their bodies. As a consequence, the virus undergoes evolutionary changes, leading to an expansion of the transmission chain.

Furthermore, the inefficient applications and institution of the One-health paradigm through the instrumentality of participatory epidemiology is another concern. The concept of participatory epidemiology serves as an integral tool in the One-health paradigm that allows for active participation of the community dwellers to collaborate with health professionals and government agencies and institutions in diagnosing, preventing and controlling infectious and non-infectious diseases. The effectiveness of this tool is mostly hinged on trust of the community stakeholders in the government and healthcare providers and professionals in providing the best healthcare services. The concept among many other advantages promotes health and wellbeing of all the general public, and establishment of a robust and effective relationship between government and communities.

## 6 Conclusion and recommendation

The need for an integrated and unifying approach toward addressing the numerous emerging infectious diseases in our world today cannot be over-emphasized. The One Health approach is increasingly considered to be the most effective policy for curtailing disease threats (7) largely due to the fact it captures a realization of certain facts about the nature and transmission of infections and development of diseases, which are then put into consideration to structure a response. The fact that human, animals, and an integrated environmental health are interdependent is now a consensus in global health management (58). We now know that several animal species constitute a shared reservoir for pathogen transmission, persistence and spread, and that many re-emerging diseases like mpox virus are driven by a very dynamic human-animal interactions (58). The biggest advantage of the One Health response to disease emergence or outbreak is the deconstruction of the restriction posed by individual health and environmental science disciplines in order to bolster the study of animal and human disease. The One Health approach has the potential to produce a creative, efficient, and sustainable solutions necessary for prevention and control of mpox virus (59). This requires an Inter-disciplinary research and surveillance effort at the local, national, and international stages with the active participation of epidemiologists, physicians, veterinarians, ecologists, environmental health officers, virologist, and more importantly policy makers (31, 32).

Also, it is important to establish efficient prevention and preparedness strategies. To mitigate the transmission and dissemination of MPXV, it is significant for the populace to refrain from any form of contact with animals or individuals displaying signs of infection. Additionally, it is crucial to consistently adhere to proper hygiene practices, promptly isolate individuals suspected of being infected, and utilize appropriate personal protective equipment when providing care to patients. In spite of the implementation of various preventive measures, there remains a possibility of an epidemic.

The inadequate of comprehensive data regarding the epidemiology of MPXV underscores the importance and advantages of doing research in any given nation. The study of the virus enables researchers and professionals to gain a more comprehensive grasp of its characteristics, including its modes of transmission and its genetic variants. Conducting research would yield significant benefits, as it would provide enhanced understanding of the virus and potentially facilitate the development of a diagnostic approach capable of distinguishing between them.

Scientists would possess the capability to predict and make necessary arrangements for any form of mutation. The research endeavors would contribute to the advancement of vaccine and antiviral manufacture. Scientists were disturbed by the recent occurrence of the MPXV in distinct populations across several regions worldwide, where its presence is often uncommon. Further research is warranted to investigate the extent of genetic variation between the strain responsible for the ongoing outbreaks and the strain observed in West Africa, as well as to ascertain the potential existence of a singular source for each of the outbreaks. The answers to these inquiries could aid in ascertaining whether the abrupt increase in cases is attributable to a mutation that enhances the transmissibility of Monkeypox compared to previous occurrences. Furthermore, the identification of MPXV in individuals lacking any documented exposure to suspected or confirmed cases implies the potential occurrence of covert transmission of the virus.

The ability to spread information is crucial for the implementation of protective measures. It is imperative that these policies be implemented across all cities within the nation, encompassing not simply major urban centers. It is imperative for urban areas to possess the capacity to independently combat and confine the spread of an epidemic. Implementing this measure would significantly decrease the likelihood of viral transmission, thereby limiting the extent of its spread. In the context of combating an epidemic, it is imperative that countries possess equitable access to resources, encompassing human capital, material provisions, and expertise. The early reaction of a nation to an epidemic or outbreak can significantly influence the economic, population effects, and duration of the outbreak.

In such a situation, it becomes necessary to devise a range of orientations, measures, and preparedness strategies. Healthcare professionals have the option to employ a technique known as “ring-vaccination” as an alternative approach to immunize individuals who have had close contact with confirmed cases of Monkeypox. This strategy aims to disrupt transmission chains and effectively confine the spread of the virus. Investing in an efficient healthcare system is a crucial aspect of readiness. Enhancing and maintaining resources allocated to healthcare budgetary provisions is a crucial strategy for fostering a well-prepared response. The allocation of funds would facilitate the enhancement of community preparedness and the provision of training for first responders and healthcare professionals. How enough the financial resources are, the more the specific sectors that require strengthening would be.

In juxtaposing the NCDC recommendations with WHO’s, early detection and containment of mpox especially in providing interventions such as therapeutics and vaccination, there is need for trust from the community. The trust is mostly achievable for the delivery, and administration of vaccines among uneducated individuals by a participatory epidemiological approach involving active involvement in the process of interventions among both the healthcare providers and the community stakeholders. Participatory epidemiology has been demonstrated to be effective in several disease outbreaks such as African sleeping sickness and tuberculosis. Participatory epidemiology is key as vaccination is a vital factor in the successful mitigation of diphtheria in the human population. Travel restriction imposed by national establishment and authorities will also help in containing the further spread of diphtheria to naïve environment, and the administration of booster vaccine to individuals that has to travel will be helpful (43).

In conclusion, the current efforts of the NCDC as well as the interventions from WHO would be successful in mitigating the further spread of mpox in Nigeria and in extension to other parts of the world when there is active participation of the community dwellers. The active involvement of individuals in trusting the healthcare providers and the health system in Nigeria is integral in the quest to minimally reduce the further spread of diphtheria. The fear of individuals concerning the administration of vaccines should be vetoed by adequate awareness creation as well as a participatory epidemiological approach. The government of Nigeria needs more geared efforts toward investment in education and research for research and development that would be able to translate to vaccine and therapeutic development. There is a need for increasing collaboration between healthcare facilities and institutions of learning and research within and outside the country. Importantly, there is a need for increasing global support from WHO and developed nations in terms of vaccine development and therapeutic development channeled toward institutional research laboratories and health facilities.

## Data availability statement

The original contributions presented in the study are included in the article/supplementary material, further inquiries can be directed to the corresponding author.

## Author contributions

SO: Conceptualization, Writing – original draft, Writing – review & editing. OCA: Conceptualization, Writing – review & editing. AA: Writing – review & editing. MMO: Writing – review & editing. DG: Writing – original draft, Writing – review & editing. MO: Writing – original draft, Writing – review & editing. RA: Writing – original draft, Writing – review & editing. JG: Writing – review & editing. BO: Writing – review & editing. ML: Writing – review & editing. ABB: Writing – review & editing. OA: Writing – review & editing. ECC: Writing – review & editing.
